# Poly[{μ_3_-3-[4-(1*H*-imidazol-1-yl­methyl)phen­yl]prop-2-enoato-κ*N*:η^2^:κ*O*}copper(I)]

**DOI:** 10.1107/S1600536811015005

**Published:** 2011-04-29

**Authors:** Benyong Lou

**Affiliations:** a Department of Chemistry and Chemical Engineering, Minjiang University, Fuzhou 350108, People’s Republic of China

## Abstract

In the coordination polymer, [Cu^I^(C_13_H_11_N_2_O_2_)]_*n*_, the Cu^I^ atom exists in a trigonal–planar geometry that is defined by the C=C unit, the imidazole N atom and carboxyl­ate O atoms from three different ozagrel ligands, resulting in the formation of a three-dimensional framework.

## Related literature

For background to the design and construction of coordination polymers, see: Kitagawa *et al.* (2004 ▶); Zhao *et al.* (2008 ▶). For other olefin complexes, see: Kato *et al.* (1997 ▶); Wang *et al.* (2005 ▶, 2007 ▶); Young *et al.* (1998 ▶); Zhang *et al.* (2001 ▶). 
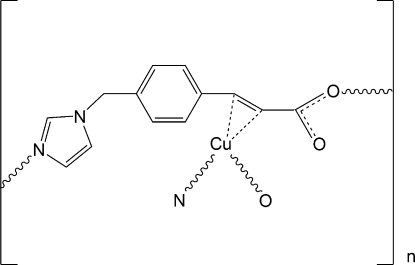


## Experimental

### 

#### Crystal data

[Cu(C_13_H_11_N_2_O_2_)]
*M* *_r_* = 290.78
Trigonal, 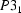

*a* = 9.7894 (19) Å
*c* = 10.483 (2) Å
*V* = 870.0 (3) Å^3^
*Z* = 3
Mo *K*α radiation
μ = 1.88 mm^−1^
*T* = 293 K
0.20 × 0.20 × 0.20 mm

#### Data collection

Rigaku Mercury CCD diffractometer
Absorption correction: multi-scan (*CrystalClear*; Rigaku, 2000 ▶) *T*_min_ = 0.765, *T*_max_ = 1.000
6852 measured reflections
2105 independent reflections
1904 reflections with *I* > 2σ(*I*)
*R*_int_ = 0.053

#### Refinement

*R*[*F* ^2^ > 2σ(*F* ^2^)] = 0.042
*wR*(*F* ^2^) = 0.105
*S* = 1.03
2105 reflections
163 parameters
1 restraint
H-atom parameters constrained
Δρ_max_ = 0.36 e Å^−3^
Δρ_min_ = −0.32 e Å^−3^
Absolute structure: Flack (1983 ▶), 773 Friedel pairs
Flack parameter: 0.05 (3)

### 

Data collection: *CrystalClear* (Rigaku, 2000 ▶); cell refinement: *CrystalClear*; data reduction: *CrystalClear*; program(s) used to solve structure: *SHELXS97* (Sheldrick, 2008 ▶); program(s) used to refine structure: *SHELXL97* (Sheldrick, 2008 ▶); molecular graphics: *SHELXTL* (Sheldrick, 2008 ▶); software used to prepare material for publication: *SHELXL97*.

## Supplementary Material

Crystal structure: contains datablocks I, global. DOI: 10.1107/S1600536811015005/ng5155sup1.cif

Structure factors: contains datablocks I. DOI: 10.1107/S1600536811015005/ng5155Isup2.hkl

Additional supplementary materials:  crystallographic information; 3D view; checkCIF report

## Figures and Tables

**Table:** 

Cu1—N1^i^	1.962 (5)
Cu1—C2	2.000 (6)
Cu1—O2^ii^	2.007 (4)
Cu1—C3	2.030 (5)
C2—C3	1.381 (7)

**Table:** 

N1^i^—Cu1—C2	151.2 (2)
N1^i^—Cu1—O2^ii^	104.12 (19)
C2—Cu1—O2^ii^	104.49 (19)
N1^i^—Cu1—C3	111.1 (2)
C2—Cu1—C3	40.1 (2)

Symmetry codes: (i) 
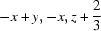
; (ii) 
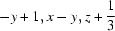
.

**Fig. 1.**
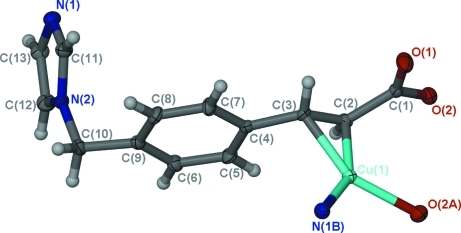
ORTEP of compound I with 30% thermal ellipsoids. A = 1-Y,X—Y,Z+1/3; B = –X+Y,-X,Z+2/3

**Fig. 2.**
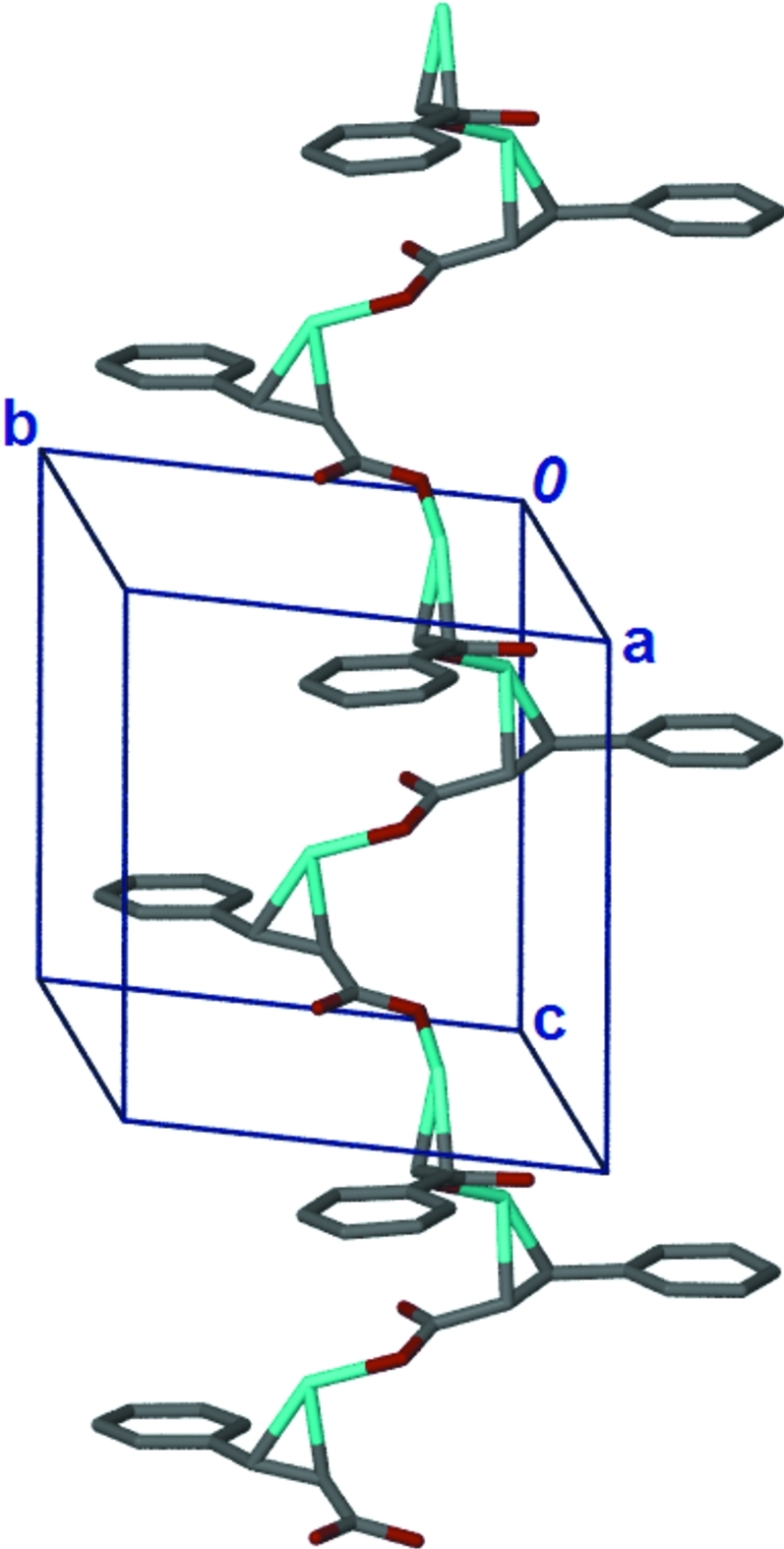
One-dimensional helical chain of Cu(I) along c axis in compound I. Imidazole group of ozagrel is omitted for clarity.

**Fig. 3.**
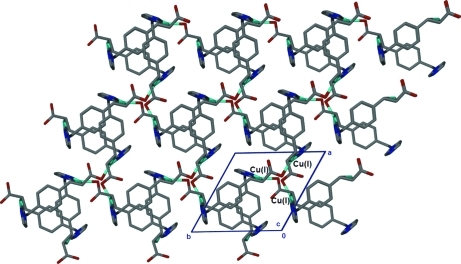
The three-dimensional structure of compound I viewed along c axis.

## supplementary crystallographic information

### Comment

The design and construction of coordination polymers have been an area of explosive growth in recent years (Kitagawa *et al.*, 2004). Some active pharmaceutical ingredients (APIs), which contain carboxylic group, N-containing ring in the structures, have also utilized for constructing specific functional coordination polymers (Zhao *et al.*, 2008). The hydrophilic or hydrophobic groups in drug molecules may play an important role in the structures and properties of final metal- organic frameworks.

Ozagrel, (*E*)-3-[4-(1*H*-imidiazol-1-ylmethyl)phenyl]-2-propenic acid, is a selective thromboxane A2-synthetase inhibitor which is used for treating cerebrovascular disease (Kato *et al.*, 1997). It has a carboxylic group and an imidazole ring in the structure. The molecule is an ideal building block for constructing coordination polymers with specific structures. In this contribution, we report a Cu(I)-olefin coordination polymer of ozagrel, [(C_13_H_11_N_2_O_2_)Cu(I)] (I), which was obtained under solvothermal reaction conditions. In the structure, conjugated olefinic and carboxylic groups of ozagrel link metal centers into a 3-fold helical chain which is linked into a three-dimensional framework structure by metal- imidazole coordination interactions.

Compound I crystallizes in the space group P31 with a deprotonated ozagrel anion and a Cu(I) cation in the asymmetric unit (Fig.1). There exist obvious interactions between Cu(I) center and C═C moiety of the olefin of ozagrel (Cu1—C2, Cu1—C3, Table 1). The C═C bond distance (1.381 Å) of the coordinated olefin is longer than that in free ozagrel (1.324 Å) (Wang *et al.*, 2007). The lengthening of the C═C distance is typical for ethylene that is η^2^-bonded to low-valent, electron-rich, transition metals such as copper(I) (Young *et al.*, 1998). Cu(I) ion is nearly centered in a trigonal planar geometry, which is defined by C═C moiety, imidazole N atom and carboxylic O atom from three different ozagrel molecules. Interestingly, carboxylic group of ozagrel doesn't serve as bidentate moiety as does it in [Cu(3-PYA)]n reported previously (Zhang *et al.*, 2001). But, conjugated olefinic and carboxylic groups as bidentate spacer link Cu(I) centers into a 3-fold helical chain along *c* axis (Fig.2). Cu(I)-imidazole interactions further link the one-dimensional helical chain into a three-dimensional framework structure (Fig.3). Thus, ozagrel anion acting as a tridentate linker is coordinated to Cu(I) ion generating a three-dimensional coordination polymer based on one-dimensional helical chain of Cu(I) centers.

Since Schultz synthesized the first air-stable Cu(I)-olefin coordination polymer based on fumarate ligand under hydrothermal conditions (Young *et al.*, 1998), some Cu(I)-olefin complexes with extended framework structures have been prepared by crystal engineering strategies (Wang *et al.*, 2005). Impressively, two luminescent two-dimensional layered copper(I)-olefin coordination polymers were constructed by the use of 3(2)-pyridylacrylic acid as tetradentate linkers (Zhang *et al.*, 2001). Therein, acrylic acid anions linked Cu(I) centers into a one-dimensional chain which was further linked into two-dimensional coordination layers by coordinated pyridyl rings. Otherwise from that in pyridylacrylic acid, the acrylic acid anion in ozagrel acts as a bidentate spacer and links Cu(I) centers into a 3-fold helical chain which is further linked into a three-dimensional framework structure by coordinated imidazole ring. In other words, rigid 3(2)-pyridylacrylic acid resulted in two-dimensional coordination layers by metal coordination to Cu(I) ion while more flexible ozagrel gave rise to a three-dimensional coordination framework. The flexible molecular structure of ozagrel could play the subtle role in the final extended structure.

In conclusion, a Cu(I)-olefin coordination polymer based on ozagrel ligand was synthesized under solvothermal conditions. Conjugated olefinic and carboxylic groups of ozagrel as bidentate spacer link Cu(I) centers into a 3-fold helical chain which is linked into a three-dimensional framework structure by metal-imidazole coordination interactions.

### Experimental

Ozagrel (228 mg, 1 mmol) and Cu(NO_3_)_2_.3H_2_O (240 mg, 1 mmol) were suspended in 10 ml me thanol and a few drops of triethylamine were added. The mixture was placed in a 23 ml Teflon-lined autoclave, sealed, and placed in a furnace at 130 °C for 2 days. Yellow block crystals were isolated. Element analysis for C_13_H_11_N_2_O_2_ Cu_1_ (%), Calcd: C, 53.65; H, 3.22; N, 9.63; Found: C, 53.57; H, 3.89; N, 9.66.

### Refinement

H atoms were located geometrically (C—H = 0.95–1.00 Å) with *U*_iso_(H) = 1.2 *U*_eq_(C).

### Crystal data

**Table:**

[Cu(C_13_H_11_N_2_O_2_)]	*D*_x_ = 1.665 Mg m^−^^3^
*M**_r_* = 290.78	Mo *K*α radiation, λ = 0.71073 Å
Trigonal, *P*3_1_	Cell parameters from 987 reflections
Hall symbol: P 31	θ = 2.4–27.4°
*a* = 9.7894 (19) Å	µ = 1.88 mm^−^^1^
*c* = 10.483 (2) Å	*T* = 293 K
*V* = 870.0 (3) Å^3^	Block, yellow
*Z* = 3	0.20 × 0.20 × 0.20 mm
*F*(000) = 444	

### Data collection

**Table:**

Rigaku Mercury CCD diffractometer	2105 independent reflections
Radiation source: fine-focus sealed tube	1904 reflections with *I* > 2σ(*I*)
graphite	*R*_int_ = 0.053
Detector resolution: 13.6612 pixels mm^-1^	θ_max_ = 27.5°, θ_min_ = 2.4°
φ and ω scans	*h* = −12→12
Absorption correction: multi-scan (*CrystalClear*; Rigaku, 2000)	*k* = −12→12
*T*_min_ = 0.765, *T*_max_ = 1.000	*l* = −9→13
6852 measured reflections	

### Refinement

**Table:**

Refinement on *F*^2^	Secondary atom site location: difference Fourier map
Least-squares matrix: full	Hydrogen site location: inferred from neighbouring sites
*R*[*F*^2^ > 2σ(*F*^2^)] = 0.042	H-atom parameters constrained
*wR*(*F*^2^) = 0.105	*w* = 1/[σ^2^(*F*_o_^2^) + (0.052*P*)^2^ + 0.5211*P*] where *P* = (*F*_o_^2^ + 2*F*_c_^2^)/3
*S* = 1.03	(Δ/σ)_max_ < 0.001
2105 reflections	Δρ_max_ = 0.36 e Å^−^^3^
163 parameters	Δρ_min_ = −0.32 e Å^−^^3^
1 restraint	Absolute structure: Flack (1983), 773 Friedel pairs
Primary atom site location: structure-invariant direct methods	Flack parameter: 0.05 (3)

### Special details

**Table:**

Geometry. All e.s.d.'s (except the e.s.d. in the dihedral angle between two l.s. planes) are estimated using the full covariance matrix. The cell e.s.d.'s are taken into account individually in the estimation of e.s.d.'s in distances, angles and torsion angles; correlations between e.s.d.'s in cell parameters are only used when they are defined by crystal symmetry. An approximate (isotropic) treatment of cell e.s.d.'s is used for estimating e.s.d.'s involving l.s. planes.
Refinement. Refinement of *F*^2^ against ALL reflections. The weighted *R*-factor *wR* and goodness of fit *S* are based on *F*^2^, conventional *R*-factors *R* are based on *F*, with *F* set to zero for negative *F*^2^. The threshold expression of *F*^2^ > σ(*F*^2^) is used only for calculating *R*-factors(gt) *etc*. and is not relevant to the choice of reflections for refinement. *R*-factors based on *F*^2^ are statistically about twice as large as those based on *F*, and *R*- factors based on ALL data will be even larger.

### Fractional atomic coordinates and isotropic or equivalent isotropic displacement parameters (Å^2^)

**Table:**

	*x*	*y*	*z*	*U*_iso_*/*U*_eq_	
Cu1	0.44797 (7)	0.33783 (7)	0.62232 (6)	0.03118 (17)	
O1	0.6474 (5)	0.5593 (5)	0.3892 (5)	0.0532 (12)	
N1	−0.3953 (6)	−0.0278 (5)	0.1055 (5)	0.0353 (11)	
C1	0.6055 (6)	0.4173 (6)	0.3901 (5)	0.0335 (11)	
O2	0.6941 (4)	0.3626 (5)	0.3546 (4)	0.0413 (9)	
N2	−0.3899 (5)	−0.0886 (6)	0.3067 (5)	0.0375 (10)	
C2	0.4448 (6)	0.2963 (6)	0.4352 (5)	0.0312 (11)	
H2	0.4069	0.1847	0.4093	0.037*	
C3	0.3305 (6)	0.3354 (6)	0.4620 (5)	0.0330 (12)	
H3	0.3597	0.4436	0.4320	0.040*	
C4	0.1578 (6)	0.2269 (6)	0.4662 (5)	0.0311 (11)	
C5	0.0875 (7)	0.0622 (7)	0.4627 (6)	0.0403 (13)	
H5	0.1519	0.0150	0.4631	0.048*	
C6	−0.0749 (7)	−0.0321 (7)	0.4588 (6)	0.0406 (13)	
H6	−0.1213	−0.1436	0.4558	0.049*	
C7	0.0604 (6)	0.2919 (7)	0.4678 (6)	0.0387 (13)	
H7	0.1061	0.4034	0.4692	0.046*	
C8	−0.1017 (7)	0.1980 (7)	0.4675 (6)	0.0417 (14)	
H8	−0.1662	0.2449	0.4731	0.050*	
C9	−0.1707 (6)	0.0355 (7)	0.4591 (6)	0.0357 (12)	
C10	−0.3482 (7)	−0.0695 (9)	0.4438 (6)	0.0441 (15)	
H10A	−0.3824	−0.1740	0.4824	0.053*	
H10B	−0.4031	−0.0216	0.4884	0.053*	
C11	−0.3627 (7)	0.0271 (7)	0.2233 (6)	0.0403 (13)	
H11	−0.3250	0.1340	0.2462	0.048*	
C12	−0.4460 (7)	−0.2242 (7)	0.2378 (6)	0.0449 (14)	
H12	−0.4761	−0.3261	0.2701	0.054*	
C13	−0.4504 (7)	−0.1860 (7)	0.1143 (6)	0.0392 (13)	
H13	−0.4863	−0.2579	0.0448	0.047*	

### Atomic displacement parameters (Å^2^)

**Table:**

	*U*^11^	*U*^22^	*U*^33^	*U*^12^	*U*^13^	*U*^23^
Cu1	0.0309 (3)	0.0364 (4)	0.0284 (3)	0.0185 (3)	0.0010 (3)	0.0030 (3)
O1	0.038 (2)	0.035 (2)	0.082 (4)	0.0155 (18)	0.004 (2)	0.012 (2)
N1	0.041 (3)	0.030 (2)	0.036 (3)	0.019 (2)	−0.003 (2)	0.002 (2)
C1	0.029 (2)	0.043 (3)	0.029 (3)	0.018 (2)	0.002 (2)	0.005 (2)
O2	0.037 (2)	0.044 (2)	0.043 (2)	0.0197 (17)	0.0039 (17)	0.0035 (18)
N2	0.030 (2)	0.044 (3)	0.035 (3)	0.016 (2)	−0.0026 (19)	0.006 (2)
C2	0.030 (3)	0.029 (2)	0.030 (3)	0.011 (2)	0.002 (2)	0.003 (2)
C3	0.031 (3)	0.041 (3)	0.030 (3)	0.021 (2)	−0.005 (2)	0.005 (2)
C4	0.032 (3)	0.033 (3)	0.028 (3)	0.016 (2)	−0.002 (2)	0.005 (2)
C5	0.041 (3)	0.047 (3)	0.043 (3)	0.030 (3)	−0.002 (3)	0.005 (3)
C6	0.036 (3)	0.036 (3)	0.042 (3)	0.012 (2)	−0.001 (2)	0.002 (2)
C7	0.029 (3)	0.031 (3)	0.052 (4)	0.011 (2)	−0.006 (2)	−0.003 (3)
C8	0.036 (3)	0.051 (3)	0.048 (4)	0.029 (3)	−0.007 (3)	0.001 (3)
C9	0.031 (3)	0.041 (3)	0.027 (3)	0.013 (2)	0.000 (2)	0.005 (2)
C10	0.030 (3)	0.054 (4)	0.036 (3)	0.012 (3)	0.001 (2)	0.008 (3)
C11	0.041 (3)	0.037 (3)	0.037 (3)	0.015 (2)	−0.002 (2)	0.004 (2)
C12	0.050 (3)	0.038 (3)	0.048 (4)	0.023 (3)	−0.002 (3)	0.000 (3)
C13	0.047 (3)	0.031 (3)	0.041 (3)	0.021 (3)	−0.003 (3)	−0.003 (2)

### Geometric parameters (Å, °)

**Table:**

Cu1—N1^i^	1.962 (5)	C4—C7	1.386 (8)
Cu1—C2	2.000 (6)	C4—C5	1.402 (8)
Cu1—O2^ii^	2.007 (4)	C5—C6	1.383 (8)
Cu1—C3	2.030 (5)	C5—H5	0.9500
O1—C1	1.237 (7)	C6—C9	1.392 (8)
N1—C11	1.320 (8)	C6—H6	0.9500
N1—C13	1.364 (7)	C7—C8	1.380 (7)
N1—Cu1^iii^	1.962 (5)	C7—H7	0.9500
C1—O2	1.281 (6)	C8—C9	1.386 (8)
C1—C2	1.496 (7)	C8—H8	0.9500
O2—Cu1^iv^	2.007 (4)	C9—C10	1.521 (8)
N2—C11	1.347 (7)	C10—H10A	0.9900
N2—C12	1.363 (8)	C10—H10B	0.9900
N2—C10	1.480 (8)	C11—H11	0.9500
C2—C3	1.381 (7)	C12—C13	1.354 (9)
C2—H2	1.0000	C12—H12	0.9500
C3—C4	1.481 (7)	C13—H13	0.9500
C3—H3	1.0000		
			
N1^i^—Cu1—C2	151.2 (2)	C6—C5—C4	120.5 (5)
N1^i^—Cu1—O2^ii^	104.12 (19)	C6—C5—H5	119.7
C2—Cu1—O2^ii^	104.49 (19)	C4—C5—H5	119.7
N1^i^—Cu1—C3	111.1 (2)	C5—C6—C9	120.3 (5)
C2—Cu1—C3	40.1 (2)	C5—C6—H6	119.8
O2^ii^—Cu1—C3	144.1 (2)	C9—C6—H6	119.8
C11—N1—C13	106.1 (5)	C8—C7—C4	121.3 (5)
C11—N1—Cu1^iii^	122.7 (4)	C8—C7—H7	119.3
C13—N1—Cu1^iii^	130.5 (4)	C4—C7—H7	119.3
O1—C1—O2	123.6 (5)	C7—C8—C9	120.2 (5)
O1—C1—C2	121.2 (5)	C7—C8—H8	119.9
O2—C1—C2	115.2 (5)	C9—C8—H8	119.9
C1—O2—Cu1^iv^	104.3 (3)	C8—C9—C6	119.2 (5)
C11—N2—C12	106.8 (5)	C8—C9—C10	121.3 (5)
C11—N2—C10	126.7 (5)	C6—C9—C10	119.5 (5)
C12—N2—C10	126.1 (5)	N2—C10—C9	109.7 (5)
C3—C2—C1	121.5 (5)	N2—C10—H10A	109.7
C3—C2—Cu1	71.1 (3)	C9—C10—H10A	109.7
C1—C2—Cu1	104.2 (3)	N2—C10—H10B	109.7
C3—C2—H2	116.6	C9—C10—H10B	109.7
C1—C2—H2	116.6	H10A—C10—H10B	108.2
Cu1—C2—H2	116.6	N1—C11—N2	111.0 (5)
C2—C3—C4	126.9 (5)	N1—C11—H11	124.5
C2—C3—Cu1	68.8 (3)	N2—C11—H11	124.5
C4—C3—Cu1	114.9 (4)	C13—C12—N2	106.9 (5)
C2—C3—H3	112.8	C13—C12—H12	126.6
C4—C3—H3	112.8	N2—C12—H12	126.6
Cu1—C3—H3	112.8	C12—C13—N1	109.2 (5)
C7—C4—C5	118.2 (5)	C12—C13—H13	125.4
C7—C4—C3	118.2 (5)	N1—C13—H13	125.4
C5—C4—C3	123.5 (5)		

Symmetry codes: (i) −*x*+*y*, −*x*, *z*+2/3; (ii) −*y*+1, *x*−*y*, *z*+1/3; (iii) −*y*, *x*−*y*, *z*−2/3; (iv) −*x*+*y*+1, −*x*+1, *z*−1/3.

## References

[bb1] Flack, H. D. (1983). *Acta Cryst.* A**39**, 876–881.

[bb2] Kato, H., Emura, S., Takeuchi, N., Enoki, M., Oogushi, K., Takashima, T., Ohmori, K. & Saito, I. (1997). *J. Int. Med. Res.* **25**, 108–111. 10.1177/030006059702500208 9100166

[bb3] Kitagawa, S., Kitaura, R. & Noro, S. (2004). *Angew. Chem. Int. Ed.* **43**, 2334–2375. 10.1002/anie.200300610 15114565

[bb4] Rigaku (2000). *CrystalClear* Rigaku Corporation, Tokyo, Japan.

[bb5] Sheldrick, G. M. (2008). *Acta Cryst.* A**64**, 112–122. 10.1107/S0108767307043930 18156677

[bb6] Wang, Y. T., Tang, G. M., Liu, Z. M. & Yi, X. H. (2007). *Cryst. Growth Des.* **7**, 2272–2275.

[bb7] Wang, X. S., Zhao, H., Li, Y. H., Xiong, R. G. & You, X. Z. (2005). *Top. Catal.* **35**, 43–61.

[bb8] Young, D. M., Geiser, U., Schultz, A. J. & Wang, H. H. (1998). *J. Am. Chem. Soc.* **120**, 1331–1332.

[bb9] Zhang, J., Xiong, R. G., Chen, X. T., Che, C. M., Xue, Z. L. & You, X. Z. (2001). *Organometallics*, **20**, 4118–4121.

[bb10] Zhao, J., Mi, L., Hu, J., Hou, H. & Fan, Y. (2008). *J. Am. Chem. Soc.* **130**, 15222–15223. 10.1021/ja8007227 18939798

